# The impact of leprosy, podoconiosis and lymphatic filariasis on family quality of life: A qualitative study in Northwest Ethiopia

**DOI:** 10.1371/journal.pntd.0008173

**Published:** 2020-03-10

**Authors:** Anna T. van ‘t Noordende, Moges Wubie Aycheh, Alice Schippers

**Affiliations:** 1 Disability Studies in the Netherlands, Almere, The Netherlands; 2 NLR, Amsterdam, The Netherlands; 3 Erasmus MC, University Medical Center Rotterdam, Rotterdam, The Netherlands; 4 Debre Markos University, Debre Markos, Ethiopia; 5 Amsterdam University Medical Centre, Location VU Medical Center, Amsterdam, The Netherlands; Swiss Tropical and Public Health Institute, SWITZERLAND

## Abstract

**Background:**

Several studies have shown that leprosy, podoconiosis and lymphatic filariasis impact individual quality of life. In contrast, family quality of life has not received as much attention despite evidence that families are also affected. This is especially relevant given the crucial role of the family in most societies around the world. This study looks at the impact of leprosy, podoconiosis and lymphatic filariasis on family quality of life.

**Methodology:**

The study used a cross-sectional design with a qualitative approach. Both semi-structured interviews and focus group discussions were conducted. Participants, persons affected and their family members, were selected by purposive sampling. Data were collected between August and November 2017 in Awi zone, Northwest Ethiopia and analysed by three independent researchers using open, inductive coding and content analysis.

**Results:**

A total of 86 participants were included in this study: 56 participants in the in-depth interviews and 30 participants in the focus group discussions. We found that participation restrictions, reduced productivity and marginalisation were common. In addition, discrimination in the communities occurred often, often extending to family members of persons affected. Divorce and difficulties in finding a spouse were common for persons affected and their family members. Many persons affected reported mental health problems. While most people got social and physical support from their families, there were a few exceptions. In particular, persons with younger children seemed to lack social support. Having to provide for their affected family member sometimes caused stress, school dropouts and an additional workload. Financial problems and loss of livelihood were reported by almost all participants.

**Conclusion:**

This study revealed that leprosy, lymphatic filariasis and podoconiosis have an effect on several dimensions of family quality of life. Many problems reported related to stigma and poverty.

## Author summary

Several studies have shown that leprosy, podoconiosis and lymphatic filariasis impact individual quality of life. In contrast, family quality of life has not received as much attention despite evidence that families are also affected. This is especially relevant given the crucial role of the family in most societies around the world. This study looked at the impact of leprosy, podoconiosis and lymphatic filariasis on family quality of life in Northwest Ethiopia. The study used a cross-sectional design with a qualitative approach. Both semi-structured interviews and focus group discussions were conducted with persons affected and their family members. A total of 86 participants were included in this study: 56 participants in the in-depth interviews and 30 participants in the focus group discussions. This study revealed that leprosy, lymphatic filariasis and podoconiosis have an effect on family quality of life, resulting in: participation restrictions, reduced productivity, marginalisation, discrimination, divorce, difficulties finding a spouse, school dropouts, mental health problems and poverty. Both persons affected and their family members were impacted. Many problems reported related to impairments, stigma and poverty. The results of this study can be used to inform leprosy, lymphatic filariasis and podoconiosis treatment and after care programmes.

## Introduction

Leprosy and lymphatic filariasis are both communicable diseases. Leprosy is caused by *Mycobacterium leprae* and is transmitted by prolonged close contact between untreated leprosy patients and susceptible and genetically predisposed individuals [1,2]. Lymphatic filariasis, sometimes called ‘elephantiasis’, is caused by three nematode worms–*Wuchereria bancrofti*, *Brugia malayi* and *Brugia timori*. The parasites that cause lymphatic filariasis are transmitted by mosquitoes [3]. Podoconiosis, or non-filarial elephantiasis, is a non-communicable disease that is believed to be caused by chronic exposure to mineral particles in red clay volcanic soil that penetrate the skin and induce an inflammatory reaction in the lymphatic system [4]. Podoconiosis predominantly affects individuals who live and work barefoot on red clay soil [4–6].

Leprosy primarily affects the peripheral nerves and skin. Nerve damage may cause impairments to their sensory, motor and autonomic functions. This may manifest itself in loss of sensation, impairments to the eyes and shortened or deformed fingers and toes [1,2]. Podoconiosis and lymphatic filariasis are both characterised by lymphoedema of the limbs, leading to enlarged legs, male genitals and female breasts [7–9]. Podoconiosis is clinically distinguished from lymphatic filariasis through commonly being ascending and bilateral but asymmetric, while lymphatic filariasis is often unilateral [7,10]. All three diseases may cause both temporary and permanent long-term impairments [1,2,8,9].

Leprosy, lymphatic filariasis and podoconiosis can have a significant social impact. Persons affected often face stigma, discrimination and social participation restrictions such as isolation, barriers to employment, education or marriage [11–20]. Living with a person affected by leprosy, podoconiosis and lymphatic filariasis can have profound effects on all family members. Often family members also experience stigma on account of having an affected person in the family [21–24]. Living with an affected person can impact many aspects of family life, such as family income and the chance of finding a marriage partner for a son or daughter [15–17,25].

Several studies showed leprosy, podoconiosis and lymphatic filariasis to have an impact on quality of life of the person affected [26–31]. In contrast, the quality of life of family members has not received much attention, despite evidence that families are also affected and play a crucial role in most societies around the world [32]. Individual quality of life broadly encompasses an individual’s perception of the ‘goodness’ of multiple aspects of their life, such as mental, physical, role, environment and social functioning [33]. Family quality of life, a natural extension of individual quality of life, is not focused on individuals but rather on all family members in the family unit [34].

Social relationships and social support play a key role in a person’s health and mental wellbeing. This is especially true for persons with physical disabilities, who due to restrictions in social participation, often have fewer opportunities for positive exchange within their close social environment [35]. High quality relationships can in turn strengthen mental wellbeing [35].

There have been several studies into the quality of life of persons affected by leprosy, podoconiosis and lymphatic filariasis [26–31,36]. However, to date no studies have been conducted on the impact of these conditions on family quality of life. The current, qualitative, study aimed to investigate how families with a family member affected by leprosy, podoconiosis and lymphatic filariasis experience their family life, what factors influence family quality of life and how having a family member with leprosy, podoconiosis and lymphatic filariasis-related disabilities impacts family quality of life, in the Awi zone, Ethiopia. This study is part of a larger project that aims to develop a family-based approach to support prevention and self-management of leprosy, podoconiosis and lymphatic filariasis-related disabilities in the Ethiopian context.

## Methods

### Study objectives

For most families, to achieve good family good quality of life all family members have to be “healthy, have a safe place to live, have a stable income, enjoy their lives together, have opportunities to learn and improve, benefit from their community supports and resources, and experience fulfilling social relationships with others” [37]. Family relations play an important role in the perceptions of individuals, and drives their behaviour, which is important for all families [38], including those with members affected by leprosy, podoconiosis and lymphatic filariasis.

The objective of this study was to explore the quality of life of families with a family member affected by leprosy, podoconiosis and lymphatic filariasis. Based on this exploration, the study aimed to develop a family-based approach to support prevention and self-management of leprosy, podoconiosis and lymphatic filariasis-related disabilities in the Ethiopian context. The ultimate aim of this approach is to improve the lives of the families involved in the study.

### Study design and study site

This study used a cross-sectional, non-random survey design with a qualitative approach. Both semi-structured interviews and focus group discussions were conducted. In-depth interviews and focus group discussions were chosen because no tools to assess family quality of life have been validated in Ethiopia to date. In addition, interviews allow pursuing in-depth information about a particular topic. The study was conducted in the Awi zone, one of the eleven zones in the Amhara region. The Awi zone is located in the Northwest of Ethiopia. The study included three woredas (districts): Zigem, Guagusa Shikudad (Injibara town), and Fagita Lekoma (Addis Kidam town). Leprosy, lymphatic filariasis and podoconiosis are all endemic in the Awi Zone [39–41].

### Study population and sample

Six groups of people were included in the study: (1) persons affected by leprosy; (2) persons affected by lymphatic filariasis; (3) persons affected by podoconiosis; (4) family members of persons affected by leprosy; (5) family members of persons affected by lymphatic filariasis; and (6) family members of persons affected by podoconiosis. Throughout this manuscript, we will sometimes refer to groups one to three as “persons affected” and groups four to six as “family members”.

We aimed to have semi-structured interviews with at least 10 persons from each participant group. We also aimed to conduct one focus group discussion per participant group with at least five participants in each group.

### Eligibility criteria

Participants had to live in one of the three districts included in the study. The persons affected had to be diagnosed with leprosy, lymphatic filariasis or podoconiosis and had to have visible impairments due to their condition. Family members had to live in the same household as the persons affected. Persons unwilling or unable to give informed consent, persons younger than 16 years of age and persons affected whose family members did not know of their condition were excluded.

### Sampling methods

Because we wanted to interview participants with specific characteristics, participants were selected using convenience sampling. Local health posts and organisations of persons affected in the study area were visited to check whether there were persons affected that met the inclusion criteria. A list was prepared of all eligible persons in the study area. From this list, participants were visited in their home and included based on their availability. Family members were selected by the persons affected from among those living in the same household, based on their availability. One family member per person affected was included.

### Data collection

Data were collected between August and November 2017. Cross-sectional data on participants’ daily life, family (quality of) life and what it is like to have an ‘affected’ family member were obtained using semi-structured in-depth interviews and focus group discussions. Interviews were conducted by four local health extension workers who spoke both Amharic and Agew languages. The interviewers were trained in leprosy, podoconiosis, lymphatic filariasis and interviewing techniques prior to data collection. The interview guides were pilot tested before data collection, minor revisions to the interview guide were made based on the pilot interviews. These participants were not included in the final sample. The interviews were conducted either in participants’ homes or, if they were members of a patient organisation, in a private space near the patient organisation. The in-depth interviews lasted on average 40 minutes, the focus group discussion lasted on average 90 minutes. The in-depth interviews and focus group discussions were audio recorded. A district coordinator monitored the entire process.

### Data analysis

The recordings of qualitative data were transcribed, translated to English and coded. A unique identifying number was given to all participants in advance, so sensitive personal data of participants were removed before analysis. Three rounds of coding were done: the first two rounds, conducted by two independent researchers, comprised of open, inductive coding and content analysis. Similar phrases with recurring themes that were derived from the data were coded. In the third analysis round, conducted by a third researcher, relevant domains were selected based on the themes identified in the first two rounds. All data were then analysed again and clustered together in different tables, ordered by theme: physical, psychological aspects and mental wellbeing, level of independence, environment, and social support and family relations. Microsoft Word, Microsoft Excel and Open Code 4.03 software were used to analyse the data.

### Ethical considerations

Ethical approval for this study was obtained as part of a larger project that aims to develop a family-based approach to support prevention of disabilities in the Ethiopian context. Ethical approval was obtained from the Debre Markos University Health Science College Research Review Committee. Permission to conduct the study in the region was also gained from the Awi zone (district) Health Desk office. The literacy rate is low in Ethiopia, only 51.8% of people over 15 years old are literate. (*http*:*//uis*.*unesco*.*org/en/country/et*). The literacy rate is even lower in our study sample, formed by persons with visible impairments and their family members predominantly living in rural areas. Therefore, all participants were verbally informed about the nature and objective of the study and of confidentiality of the data prior to data collection. All participants were allowed to ask questions and were given sufficient time to consider whether or not they wanted to participate in the research. Enrolment was voluntary, verbal consent from each participant was obtained prior to data collection. Parental consent was obtained in addition to the child’s own consent for the two participants who were below 18 years old.

## Results

### Demographic information

A total of 86 participants were included in this study. Fifty-six participants were included in the in-depth interviews: 14 persons affected by podoconiosis, 12 persons affected by leprosy, 12 persons affected by lymphatic filariasis, one person affected by both leprosy and lymphatic filariasis and 17 family members of persons affected. Family members were children (n = 8), spouses (n = 4), parents (n = 2), sibling (n = 2) or grandparents (n = 1). Most family members (n = 11) were family members of a person affected by podoconiosis instead of leprosy (n = 3) or lymphatic filariasis (n = 3). The average family size was 5 people, ranging from 1 to 10 (standard deviation 2.5).

The average age of all participants was 43 years (range 17–73). Persons affected by leprosy (51 years) and podoconiosis (48 years) were, on average, older than persons affected by lymphatic filariasis (39 years) and the group of family members (36 years). Many participants were farmers (n = 16) or worked in daily labour (n = 16). Three persons affected and two family members were students. Almost two-third of the participants had no formal education (n = 36).

Four focus group discussions were conducted to supplement the in-depth interviews. Thirty people were included in the focus group discussions: ten persons affected by leprosy, six children/grandchildren of persons affected by leprosy, eight persons affected by podoconiosis and six children of persons affected by podoconiosis. The average age was 52 (range 25–80) for the persons affected and 20 (range 16–27) for the family members. An overview of the number of participants in the interviews can be found in Table 1. All persons affected included in the focus group discussions were uneducated (n = 18/18), while most family members had completed primary or secondary education (n = 11/12). Table 2 provides an overview of the demographic information of the participants.

**Table 1:** Overview of the number of participants (*n* = 86) included in the in-depth interviews and focus group discussion.

	Persons affected		Family members of persons affected	
	Interview	Focus group discussion	Interview	Focus group discussion
Podoconiosis	14	8	11	6
Lymphatic filariasis	12	-	3	
Leprosy	12	10	3	6
Leprosy and lymphatic filariasis	1	-	-	-
*Total*	39	18	17	12

**Table 2:** Socio-demographic characteristics of the participants (*n* = 86).

	In-depth interviews (*n* = 56)	Focus group discussions (*n* = 30)
Average age (range)	43 (17–73)	39 (16–80)
Female, *n* (%)	32 (57%)	16 (53%)
Participant type Person affected podoconiosis, *n* (%) Person affected by leprosy, *n* (%) Person affected by LF, *n* (%) Person affected by leprosy and LF, *n* (%) Family member, *n* (%)	14 (25%) 12 (21%) 12 (21%) 1 (2%) 17 (30%)	8 (27%) 10 (33%) - - 12 (40%)
Family size, mean (range, SD)	5 (1–10, 2.5)	5 (2–7, 1.5)
No education, *n* (%)	36 (64%)	19 (63%)
Occupation Farmer, *n* (%) Daily labour, *n* (%) Student, *n* (%) Other, *n* (%)	16 (29%) 16 (29%) 5 (9%) 19 (34%)	15 (50%) 8 (27%) - 7 (23%)

### Physical: symptoms, cause and self-care

Persons affected by podoconiosis and lymphatic filariasis reported symptoms like itching, rashes and swelling–mostly of the legs. The majority of the persons affected by podoconiosis and lymphatic filariasis (n = 20/26; 11 persons affected by podoconiosis and 9 persons affected by lymphatic filariasis) reported that they frequently experienced pain because of their condition. Persons affected by leprosy described their symptoms as itching, a loss of sensation and/or not feeling pain, wounds and inability to use their hands. Some participants (n = 11/39) said their symptoms increased over time.

A person affected by leprosy explained:

*“…First it started when I swam in the river with a scabies-like rash on my whole body and it was itching*, *finally the wound started from my foot and spread to my whole body*, *then a feeling of senseless*, *finally it eats my fingers and I lose my fingers…”* (Man affected by leprosy, age 60)

Most participants in the in-depth interviews, persons affected and their family members, believed the disease was either God’s will (n = 26/56) or punishment (n = 2/56), hereditary (n = 17/56) or caused by something else (n = 9/56). Some participants (n = 7/39) indicated that the disease is normally hereditary, but not in their case because they didn’t have any relatives who were affected by the disease. Most participants in the focus group discussions believed the disease was ‘from God’, hereditary, due to sins, or because of walking barefoot. One participant explained:

*“…My father and I assumed that the disease would be transmitted to my children but the reality is not that because my children are still not affected now…”* (Man affected by leprosy, age 64, focus group discussion)

A number of participants believed traditional medicine or holy water would cure their condition. Almost a quarter of the persons affected (n = 9/39) explained they went to the holy waters to try and get a cure for their condition or some relief from the pain. Some participants said they (had) used traditional medicine (n = 6/39; 3 persons affected by podoconiosis, two lymphatic filariasis, one leprosy). The majority of the participants said they regularly practiced self-care. Two participants indicated they were not able to practice self-care because they felt weak. One participant explained:

*“…I wash my leg using soap and water and I wear my shoes*. *My family takes me to health centre and holy water…”* (Woman affected by lymphatic filariasis, age 25)

### Psychological aspects and mental wellbeing

The majority of the persons affected (n = 21/39; ten persons affected by lymphatic filariasis, five podoconiosis, five leprosy, one leprosy and lymphatic filariasis) described a mental health issue. Some participants said that they felt inferior compared to their friends or community members (n = 7/39; four persons affected by leprosy, two lymphatic filariasis, one podoconiosis). A few participants affected by podoconiosis or lymphatic filariasis said that they (n = 3/26) or their affected family member (n = 2/26) sometimes felt sad or depressed. Three other participants, women affected by lymphatic filariasis, indicated that they felt like they had no opportunities.

Other psychological challenges described by the participants include worrying (n = 3/39), low self-esteem (n = 2/39), being ashamed (n = 2/39), feeling hopeless (n = 1/39), feeling deserted (n = 1/39) and sleeping problems (n = 1/39). In addition, one person affected by leprosy from the focus group discussion said he used to have suicidal thoughts.

### Level of independence: Day-to-day life, work and resources

Over three quarters of the participants reported on their ability to move around and do their day-to-day activities (n = 30/39). One third of the participants described some (n = 8/30) or severe (n = 11/30) activity limitations. Participants said they had difficulty moving, for example they were unable to walk long distances, unable to move and/or had a low energy level. Some participants affected by podoconiosis or lymphatic filariasis (n = 5/30) indicated that they only experienced limitations when they were in pain. There were also participants who did not have any problems moving around or in their day-to-day activities (n = 6/30):

*“…I can walk*, *move and take care of myself…”* (Man affected by lymphatic filariasis, age 40)

Almost one in five persons affected said they were still able to work as they did before (n = 7/39), five persons affected did not answer and almost three quarters of the persons affected said they were not able to work as before (n = 27/39; 12 podoconiosis, nine leprosy, five lymphatic filariasis, one leprosy and lymphatic filariasis). In addition, not being able to work in the same capacity was mentioned in the focus group discussions by a number of participants (n = 9/30; three persons affected by leprosy, six family members). The participants who indicated they were not able to work as before said this was the case because of their condition, pain, disability or because working on the land had become too hard. Some participants had taken on other, lighter work (n = 5/39).

*“…I work in handicraft since my hand’s fingers are well (…) I cannot do my previous agricultural work because of my disease*. *If [I’m] exposed to soil and mud my wound aggravates*, *that makes me poor*. *I thank God my hand is well* …*”* (Woman affected by leprosy, age 56)

Almost half of the persons affected and their family members stated they were poor, lived in poverty and/or lacked money, without being asked about their financial situation by the interviewer (n = 27/56). An additional eight participants indicated they were in need of money, without mentioning that they were poor. All participants who said they went to the holy water, a costly expedition, said they were poor. Other things asked for by the participants include “being cured” or (effective) treatment (n = 21/56), materials such as Vaseline and shoes (n = 10/56), government support (n = 7/56) and a loan (n = 5/56). In addition, some participants (n = 8/56) stated that they wished to move from the rural to the urban areas, because there is less mud in the towns. One participant said:

*“…I became economically dependent on my family due to my disease (…) I wish I was cured either by holy water or drugs so that [I’m] able to work effectively*. *My great obstacle is poverty*. *I cannot afford soap*, *food and transport for my treatment…”* (Man affected by podoconiosis, age 35)

Similar results were found in the focus group discussions. In addition, participants from the focus group discussion explained that living further away from the town also brings additional costs for transportation.

### Environment: Attitudes and social participation

Discrimination in the communities was common. The majority of the participants in the in-depth interviews (n = 27/39; 11 podoconiosis, ten leprosy, five lymphatic filariasis, one leprosy and lymphatic filariasis) and almost half of the persons affected in the focus group discussions (n = 8/18) said they were discriminated against by their community members. Many of these participants (n = 20/27 in the in-depth interviews and n = 7/8 in the FGD) were also insulted, e.g. they were called ‘leper’, ‘lost finger’ or ‘swollen leg’. Two participants explained:

*“…Some of the community members see him as inferior…”* (Wife of person affected by podoconiosis, age 45)

And

*“…Many of my neighbours used to say we cannot enter their house*, *they separate me from coffee [ceremonies] too*. *Sometimes when I said hello to kids their parents were not happy*, *some of them warned me not to touch them*. *That was the worst time during my illness (…) When people discussed my disease and prevented [me] from social life my wife asked me to divorce…”* (Man affected by leprosy, age 45)

Some participants said they had no, bad or limited social contact with their neighbours and/or community members (n = 6/39; three podoconiosis, two leprosy, one lymphatic filariasis). There were also participants who had a good relationship with the community (n = 7/39; four podoconiosis, two lymphatic filariasis, one leprosy). One participant said:

*“…Like me my children also live in good relationship with the community…”* (Woman affected by podoconiosis, age 70)

Almost half of the family members of persons affected in the in-depth interviews (n = 8/17) and all the family members of persons affected by leprosy in the focus group discussions (n = 6/6) also experienced discrimination. They were either insulted or discriminated against (n = 7/17) or had to leave school because of discrimination or because they had to work to help provide for the family (n = 4/17). Two persons affected said they had trouble finding a husband or wife for their children because of their condition. Two participants explained:

*“…We have a good relation with the majority of the community but some individuals abuse us by saying ‘lost finger son’ and the like…”* (Son of person affected by leprosy, age 20, focus group discussions)

And

*“…Some people insult me and also they insult my children (…) People in the wedding made me stay outside of the tent and they did not treat me as [if] I was healthy*. *They are afraid of my disease and don’t want it to be transmitted to them*. *How can I be equal with this disease*, *I sometimes agree with what they did (…) One of my daughters went to school*, *her friends insult her (…) and now she does not go to school…”* (Woman affected by podoconiosis, age 40)

About one-third of the persons affected (n = 12/39) indicated that they didn’t experience any social participation restrictions. In addition, one-third of the persons affected indicated that they experienced social participation restrictions due to activity limitations (n = 6/39), stigma (n = 3/39), activity limitations and stigma (n = 2/39) or their physical condition (n = 2/39). Social participation restrictions included isolation, not being invited to weddings, the houses of friends and coffee ceremonies and barriers to employment and education. Two students stated they had to stop their education because they were ill.

*“…I go to church every morning and help my family with household work (…) I stopped my education due to frequent [acute] attacks of the disease…”* (Woman affected by lymphatic filariasis, age 18).

### Social support and family relations

Over half of the participants said that family support given to the affected family member was good (n = 30/56, 20 persons affected and 10 family members). One in five participants said that persons affected received some support from family members (n = 12/56; eight persons affected and four family members). An additional one in five participants said no or limited support was given to affected family members (n = 12/56; 11 persons affected and one family member). In four “participant pairs” of a person affected by podoconiosis and their family member, the family member thought they were giving more support than the person affected perceived. One family member explained:

*“…We are also in fear of contracting the disease*. *For more than one year and eight months I wash his feet*, *hands*, *take care of his urine and feed him*. *Now he starts to take care of himself*. *We took him to the holy water and the hospital but there is no change and he is not cured (…) There is nothing suitable for the poor*, *we are in trouble*. *Both our kids and I are working day-to-day as daily labour to support the family…”* (Wife of person affected by leprosy, age 36)

Support from family members included moral or psychological support, providing money or other resources, taking over household duties such as cooking and washing clothes and helping with self-care. Participants mostly relied on their children and spouse for support, calling them their “great opportunity”. One third of the persons affected were very dependent on others due to motor restrictions (n = 3/39) or because they had no or very limited social support (n = 10/39; four persons affected by podoconiosis, four lymphatic filariasis, two leprosy). The participants who indicated they had no or very limited social support were living alone, had young children, were very poor with a big family to support or divorced. One participant explained:

***“…****There is no support from my family (…) My wife is weak and cannot give me support and my children are small…”* (Man affected by lymphatic filariasis, age 40)

In the focus group discussions, the participants explained that young children can’t help their families when they are at school and that it is often difficult to afford sending children to school. For this reason, many children of affected families drop out of school early. At the same time, having to provide for their affected family member also impacts the family members, causing stress and additional workload. One participant explained:

*“…When he was healthy he supported us but because of the disease we are forced to help him rather than getting help from our father*. *We left our education in order to support the family by doing daily work…”* (Daughter of person affected by leprosy, age 18, focus group discussion)

Some participants explained that they not only needed support, but also had to give (financial) support their family members (n = 4/39). Poverty was a challenge for many families.

One in five participants were divorced because of their condition (n = 6/39) or continuously asked by their partner for divorce (n = 2/39). One participant explained:

*“…My wife repeatedly asks me to be divorce and even she was lost for more than two weeks*. *Then I begged the elders and priests in the town for her to come back*. *Especially her relatives forced her to leave me* …*”* (Man affected by leprosy, age 45)

Divorce seemed to occur more often among persons affected by leprosy (n = 5/8): three persons affected by leprosy were divorced and the spouses of two persons affected by leprosy asked for divorce. Divorce seemed to have a negative impact on mental wellbeing. One participant said:

*“…[I] divorced with my husband (…) He married another wife and had two additional children (…) It was the worst situation in the last times to live with the community but now it is improved*. *I feel ashamed when people [include] me in social interactions…”* (Woman affected by leprosy, age 56)

Many persons affected by leprosy said they were part of a leprosy association that gave them a lot of support (n = 7/12). Two participants received a lot of support from their neighbours. One participant explained:

*“…My home renter told me to leave his house since I cannot pay on time*. *I was forced to leave his house with my children*. *However*, *my neighbours pay my rent (…) my neighbours lend me money for holy water and other expenses (…) The people around me helped me what they can …”* (Man affected by leprosy, age 38)

## Discussion

This study revealed that leprosy, lymphatic filariasis and podoconiosis have an impact on several dimensions of family quality of life. That leprosy, lymphatic filariasis and podoconiosis impact individual quality of life is supported by several other studies[26–31,36]. Two quantitative studies that have been conducted on the impact of podoconiosis on individual quality of life in Ethiopia found that podoconiosis has a negative effect on individual quality of life [29,42]. The present study is the first study into family quality of life of these conditions.

We found that persons affected often experience pain due to their condition. Some participants had activity limitations, such as not being able to walk long distances and an inability to move at all. In one-third of the persons affected in our study, activity limitations and stigma led to social participation restrictions such as isolation, not being invited to coffee ceremonies and barriers to employment and education. These findings are similar to other studies conducted in Africa. In studies among persons affected by leprosy in Nigeria and Mozambique [43–45], persons affected by lymphatic filariasis in Nigeria, Ghana and Malawi [46–50], and persons affected by podoconiosis in Ethiopia [51,52], those interviewed also reported (severe) social participation restrictions. This was often linked to stigmatisation of persons affected [43–47,50,52]. In addition, in a cross-sectional survey study among 233 community members of persons affected by leprosy in Cameroon, only one-third of the participants approved of participation of persons affected by leprosy [53].

The present study also found that discrimination in the communities was common, often extending to family members of persons affected. Persons affected and their family members were sometimes socially excluded and insulted by their community members and divorce and difficulties in finding a spouse were not uncommon. Similar findings were found in a study on women with disabilities in Ethiopia, who experienced societal denial of marriage and motherhood [54]. Several studies among persons affected by leprosy, lymphatic filariasis and podoconiosis found high levels of stigma—for example among persons affected by podoconiosis in Ethiopia [17,20,29,52,55–59]. Some of these studies attributed the high levels of stigma to beliefs about the disease's causation–the belief that podoconiosis is hereditary [55,59]. This belief and the fear of costs of treatment and of disability in turn had a negative influence on marriage prospects and marital stability, also for family members of persons affected [55,59]. High levels of stigma were also found among persons affected by lymphatic filariasis in Ghana [15,50] and Nigeria [46,47] and persons affected by leprosy in Ghana [60,61], Tanzania [62] and Nigeria [43,45,63,64]. Some studies in Africa found that persons affected by leprosy and lymphatic filariasis are also stigmatized by their family members [50,61,62]. This was not found in the nuclear family in the present study.

Some studies found stigma to deteriorate the economic situation of persons affected [45,46,50]. This was found in the present study also, as almost all participants reported financial problems and loss of livelihood. In addition, in our study, almost three-quarters of the persons affected said they were not able to work as before because of their condition. A recent literature review into the extent, similarities and differences of social stigma in neglected tropical diseases found evidence that reduced work opportunities are common among persons affected by neglected tropical diseases such as leprosy, lymphatic filariasis and podoconiosis [11]. In our study, most participants who indicated they were unable to work said they were physically unable to work because of their condition. We think that in the present study, physical impairments that hamper daily functioning in productive activities, high occurrence of divorce and hence loss of social support, large families to support and high costs for (alternative) treatment exacerbated financial problems of participants.

We found that persons with younger children seemed to lack social support. Participants explained that young children can’t help their families when they are at school and that they can’t always afford to send their children to school. For this reason and because of stigma, persons affected and children of affected families drop out of school early. In addition, having to provide for their affected family member also impacted the family members, causing stress and an additional workload. These findings suggest that providing care to affected family members may result in physical, emotional, financial and social burdens that can diminish their (family) quality of life. This is supported by studies in other fields [65–68]. However, in the present study we found that most people receive social and physical support from their families. This is reflected by other studies on family quality of life where families that include a member with disabilities reported positive aspects on their family life, such as problem solving and family sense of coherence [37]. Results from worldwide research on family quality of life show that positive family relationships are a common strength of most families where one or more members have a disability [69], where negative feelings are likely to be attributed to societal norms that are imposed on families [70].

We found that half of the persons affected sometimes experienced negative affect such as feeling sad, ashamed, worried or hopeless. This is consistent with what is already known about the psychological effects of stigmatized conditions [71,72]. In the present study, some persons affected lacked social support or were dependent on others. Two literature reviews found that social relationships play a key role in mental well-being in persons with disabilities [35] and in quality of life of people with mental health problems [73]. In addition, strengthening social support can increase a person’s feeling of belonging. Connection and belonging are important to quality of life [73,74]. Some studies even suggest that people are fundamentally motivated by a need to belong [74]. This suggests that strengthening social support and quality relationships may improve mental wellbeing of persons affected by leprosy, lymphatic filariasis and podoconiosis.

Lastly, we found that the quality of life dimensions that were affected (the domains physical, psychological aspects, independence, environment, and social support and family relations) were similar among the three conditions. This finding is supported by a literature review on health-related stigma, that found that the consequences of stigma affect the quality of life of persons affected and that the areas of life affected by stigma are similar in different conditions and different cultural settings [72].

A limitation of this study is the non-random sampling and the small study size per participant group. In addition, the study focused on one geographic location only. This means that the results of the study cannot be generalized to the whole study population or beyond. Another limitation of the study is that we did not register the frequency of ‘acute attacks’ in persons affected by lymphatic filariasis and podoconiosis. We also did not register severity of disability and the occurrence of reactions in persons affected by leprosy. Acute attacks and leprosy reactions may affect the quality of life of persons affected. However, we collected data on participants’ experiences of pain.

Taking the above limitations into account, the results of our study offer insights into the impact of leprosy, lymphatic filariasis and podoconiosis on family quality of life in Awi zone, Ethiopia. The results of this study have a number of implications for leprosy, lymphatic filariasis and podoconiosis treatment and after care programmes. Many of the problems reported in the present study related to impairments, stigma and a lack of finances. A family-based approach that addresses self-care and social and economic aspects may improve individual and family quality of life. Micro-credit loans and vocational training may reduce stigma by protecting persons affected against loss of social value [63] while strengthening social support and quality relationships may improve mental wellbeing of persons affected and their family members [73,74]. Efforts to improve quality of life of persons affected and their family members should give priority to those who are living alone, have young children, or do not have a partner.

### Conclusion

This study revealed that leprosy, lymphatic filariasis and podoconiosis have an effect on several dimensions of family quality of life. Physically, persons affected often experience pain. Psychologically, persons affected experienced negative affect, for example feeling depressed, inferior, deserted, ashamed, worried and/or hopeless. Socially, participation restrictions, reduced productivity and marginalisation were common. Discrimination of persons affected and their family members occurred often. Divorce and difficulties in finding a spouse, especially for persons affected by leprosy, were not uncommon, even extending to their family members. Persons with younger children seemed to lack social support. Having to provide for their affected family member sometimes caused stress, school dropouts and an additional workload. Financial problems and loss of livelihood were reported by almost all participants.

We found that the areas of life that were affected were similar among the three conditions. This indicates that programmes focusing on treatment and after care of persons affected should follow a holistic approach that addresses the physical, psychological, social and environmental impact of the disease and focus on the entire family. A family-based approach that addresses self-care and social and economic aspects may improve individual and family quality of life. Efforts to improve quality of life of persons affected and their family members should give priority to those who are living alone, have young children, or do not have a partner.

## Supporting information

S1 Checklist STROBE Checklist. (DOC)
